# It’s not all about the Soprano: Rhinolophid bats use multiple acoustic components in echolocation pulses to discriminate between conspecifics and heterospecifics

**DOI:** 10.1371/journal.pone.0199703

**Published:** 2018-07-18

**Authors:** Robert N. V. Raw, Anna Bastian, David S. Jacobs

**Affiliations:** 1 Department of Biological Sciences, University of Cape Town, Cape Town, South Africa; 2 School of Life Sciences, University of KwaZulu-Natal, Durban, South Africa; University of Western Ontario, CANADA

## Abstract

Acoustic communication plays a pivotal role in conspecific recognition in numerous animal taxa. Vocalizations must therefore have discrete acoustic signatures to facilitate intra-specific communication and to avoid misidentification. Here we investigate the potential role of echolocation in communication in horseshoe bats. Although it has been demonstrated that echolocation can be used to discriminate among con- and hetero-specifics, the specific acoustic cues used in discrimination are still relatively unknown. Furthermore, the Acoustic Communication Hypothesis proposes that in multispecies assemblages, in which echolocation frequencies are likely to overlap, bats partition acoustic space along several dimensions so that each species occupies a discrete communication domain. Thus, multiple echolocation variables may be used in discrimination. The objective of this study was to investigate the potential of various echolocation variables to function as discriminatory cues in echolocation-based species discrimination. Using habituation–dishabituation playback experiments, we firstly tested the ability of *Rhinolophus clivosus* to discriminate between echolocation pulses of heterospecifics with either discrete or overlapping frequencies. Secondly, to determine whether *R*. *clivosus* could use echolocation variables other than frequency, we investigated its ability to discriminate among echolocation pulses differing in only one manipulated parameter. These test variables were identified by their contribution to the dissimilarity among pulses. Our results suggest that *R*. *clivosus* could discriminate readily between species using echolocation pulses with discrete frequencies. When frequencies overlapped, the ability of bats to discriminate was dependant on additional acoustic variables that defined the acoustic space occupied by the test signal. These additional acoustic variables included, but may not be restricted to, sweep rate of the FM and duty cycle. Thus, when echolocation pulses share a similar acoustic domain, bats use several cues to reliably discriminate among heterospecifics.

## Introduction

Species discrimination plays an important role in the life history of animals. In particular, the reliable identification of potential mates is vital for successful reproduction, especially in the presence of similar congeneric heterospecifics [1], resulting in strong selection pressure for discrimination of conspecifics from similar sympatric species [2]. Failure to successfully discriminate between conspecifics and heterospecifics may lead to a number of fitness costs, including wasted time and the production of unfit hybrids [3]. Animals are able to discriminate between conspecifics and heterospecifics through species-specific cues. Such cues may be conveyed by sight, odour, touch or sound or a combination of all of these. Species discrimination has, therefore, shaped the evolution of many communication signals [4]. Accordingly, acoustic signals often diverge among sympatric species into species-specific signals [4]. Almost all acoustic signals have evolved exclusively as a means of communication, with the exception of echolocation. Echolocation is a sophisticated acoustic sensory system that is used by only a few taxa, including bats, dolphins, some birds and rodents [5–7], for orientation and foraging [8]. When echolocating, the animal emits a series of sonar pulses which are reflected off objects as echoes. Differences between the emitted pulse and the returning echo provide the animal with detailed information about its environment [8].

As with all acoustic signals, bat echolocation is influenced by environmental factors [9] such as atmospheric conditions which impact on the distance the sound travels [8]. It is firmly established that the spectral (e.g. frequency with maximum energy, bandwidth) and temporal (e.g. pulse duration) characteristics of echolocation pulses are adapted for the exploitation of particular ecological niches [8,10,11] and prey types [12]. Given that many predatory bat species use similar habitats and foraging modes to exploit similar prey items, the acoustic structure of echolocation signals in different species are often convergent, shaped by the similarities in the mode of foraging and habitat, regardless of evolutionary relatedness [13,14]. Echolocation pulses may therefore not necessarily encode the unique species-specific cues that are characteristic of communication signals (see for example [15]). However, despite similar selection pressures on signal design, the acoustic structure of some echolocation pulses has been found to encode intraspecific signals carrying information about individual identity [16–20], gender [16,20–22] and colony membership [23–26], as well as interspecific signals allowing species discrimination [2,27–31]. Bat echolocation, which primarily evolved in the context of orientation and foraging, may therefore also function in communication [2,20,32–36].

**Fig 1:**
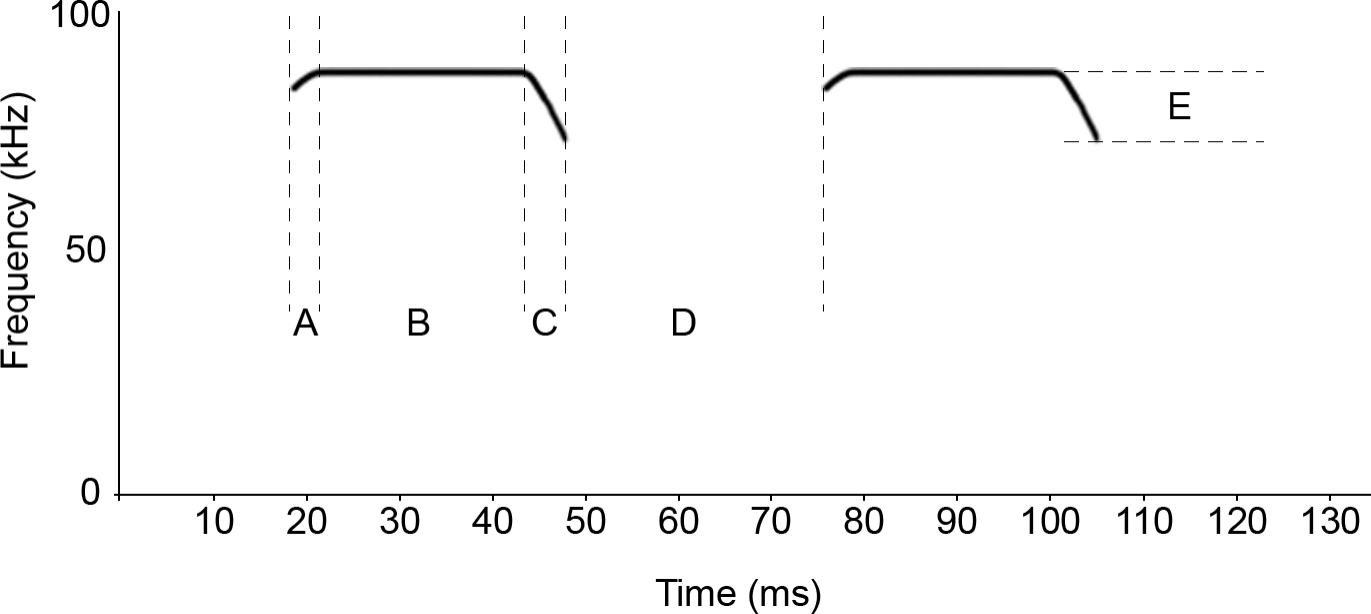
Example of a high duty cycle echolocation pulse of a horseshoe bat (*R*. *clivosus*) with a resting frequency of 92 kHz. The sonogram shows only the dominant second harmonic, first and upper harmonics as well as background noise have been excluded for clarity (for a complete example of a pulse see Finger et al. [20]). Individual pulse components are labelled–A: initial FM component. B: CF component. C: terminal FM component. D: inter pulse interval. E: bandwidth of the FM component.

Subsequent to the discovery of such “self-reporting signatures” [37] in echolocation pulses, experimental studies tested the ability of bats to perceive these signatures. Much of the work on species discrimination has been done on horseshoe bats (Rhinolophidae). Horseshoe bats are ambush hunters and echolocate readily while at rest, scanning their environment for insect prey [8,38]. They belong to the group of high duty-cycle (HDC) echolocating bats–the pulses of which are characterised by long, narrowband echolocation pulses, separated by relatively short inter-pulse intervals [8]. Rhinolophid HDC pulses are dominated by a long constant frequency (CF) component, flanked by two short frequency modulated (FM) components (Fig 1). The frequency of the CF component of these pulses emitted while at rest, referred to as the ‘resting frequency’ (RF; Fig 1), is a few hundred Hertz below that of the acoustic fovea of each bat [39]. The acoustic fovea is a disproportionately high representation of neurons in the auditory system associated with a narrow range of frequencies known as the reference frequency [40,41]. In combination with Doppler shift compensation, the acoustic fovea allows the detection of fluttering insects in dense vegetation [42]. While in flight the frequency of the echoes of the bat’s echolocation pulse will be shifted up as a result of the motion of the bat relative to a target. To compensate for this shift in the frequency of the echo, known as the Doppler effect (hence Doppler shift compensation, DSC), the bat emits echolocation pulses with a CF frequency at a slightly lower frequency than the reference frequency so that the frequency of the returning echo falls within the narrow frequency range of the acoustic fovea. Given the importance of the CF component to the echolocation system of these bats and early reports that different sympatric species appear to use different CFs [43], it was assumed that frequency of the CF component was the crucial carrier of species-specific cues.

Several studies have investigated the role of the CF frequency as a carrier of species-specific cues. For example, interspecific discrimination based on the resting frequency of echolocation pulses was demonstrated in six species of horseshoe bats–namely *Rhinolophus mehelyi*, *R*. *euryale*, *R*. *macrotis*, *R*. *lepidus*, *R*. *sinicus* and *R*. *capensis* [2,31,34]. The importance of the frequency of the CF components for species discrimination in horseshoe bats has, however, been questioned. For example, there was no correlation between frequency differences of the CF component of the echolocation pulses of sympatric conspecifics and heterospecifics and the discriminatory performance of listening *R*. *macrotis*, *R*. *lepidus* and *R*. *sinicus* [31], suggesting that additional cues in the pulses were involved in discrimination. The exclusive role of CF for species discrimination was further challenged by the results of a study by Schuchmann and Siemers [2] which showed that two species of horseshoe bats, *R*. *mehelyi* and *R*. *euryale*, were both able to discriminate between pulses of congeneric heterospecifics even when the frequencies of the CF overlapped considerably. Bastian and Jacobs [34] then demonstrated that certain populations of *R*. *capensis* discriminated echolocation pulses from heterospecific horseshoe bat species with both discrete and overlapping frequencies, whereas other populations failed to discriminate the same combinations when frequencies overlapped. They concluded that bats in more complex acoustic assemblages evolved increased perceptual acuity allowing them to discriminate echolocation pulses of hetero- and conspecifics even when their pulses overlapped in frequency by using a combination of pulse variables including the FM components. Echolocation based species discrimination would therefore be facilitated by signal divergence through the partitioning of acoustic signal space (rather than just acoustic frequency bands) of the senders and the ability of the receiver to perceive these multiparametric cues. The Acoustic Communication Hypothesis (ACH) [43–47] as reformulated by Bastian and Jacobs [34] thus proposes that multidimensional echolocation space may be partitioned in assemblages of sympatric congeneric species to facilitate species specific communication channels. Each species would occupy a distinct echolocation domain, rather than a “private frequency band” [43], facilitating echolocation based intraspecific communication and species discrimination. Although, the primary function of echolocation is likely to place limitations on the extent to which echolocation pulses can diverge in multidimensional space (see Bastian and Jacobs [34]), we still need to know which variables define this multidimensional echolocation space for discrimination. Both, Bastian & Jacobs [34] and Finger et al. [20] used a multiparametric analysis to reveal several acoustic variables that may encode vocal signatures. However, the components of echolocation pulses actually used by bats have not yet been experimentally tested.

In this study, we used playback experiments to firstly, confirm that the focal species can discriminate among conspecifics and heterospecifics based on echolocation pulses alone regardless of whether resting frequencies overlap and secondly, to determine which variables of echolocation are used by listening bats for discrimination. Our model system provided us with an opportunity to do so through a combination of experiments. The system comprised three horseshoe bat species whose CF components had overlapping frequencies in allopatry but discrete frequencies in sympatry. This allowed us to use the pulses of the same species as signals with overlapping frequencies (from an allopatric population) and discrete frequencies (from a sympatric population) to test the following predictions of the reformulated ACH [34]: a bat should be able to discriminate between pulses of its own and another species using the resting frequency or, in cases where these overlap, other acoustic cues. In our study, the use of the same species for signals with overlapping or discrete frequencies controls for the inadvertent introduction of other unidentified differences in pulses when signals with overlapping or discrete frequencies are derived from different species.

## Materials and methods

### Study animals

**Fig 2:**
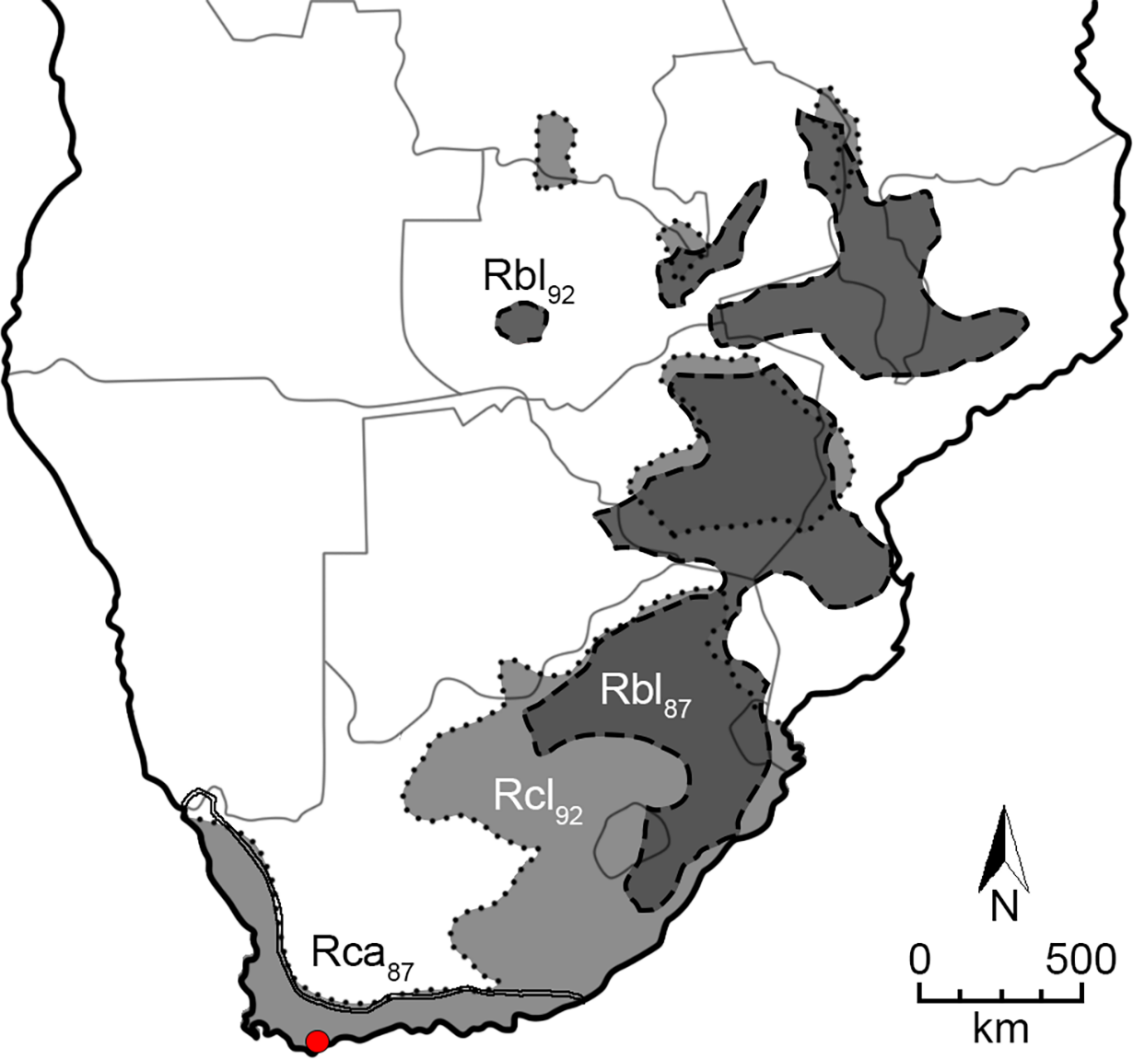
Map of southern Africa showing the distribution of the three horseshoe bat species used in this study: *R*. *clivosus* (Rcl, dotted line, light grey; [48]), *R*. *blasii* (Rbl, dashed line, dark grey; [48]) and *R*. *capensis* (Rca, solid line, white–almost completely covered by the south-eastern part of the distribution of *R*. *clivosus*; [49]). The study site in the field at De Hoop Nature Reserve, is indicated by a red dot. Numbers following the species abbreviation indicate the average resting frequencies of the three species at the location of their recording.

We used *R*. *clivosus* as a focal species to which we played back recorded echolocation pulses from two other rhinolophid species, *R*. *blasii* and *R*. *capensis*, which are sympatric with *R*. *clivosus* over most of its geographic range in southern Africa, but allopatric to each other [48] (Fig 2).

*Rhinolophus clivosus* is an insectivorous bat (forearm length (FA): 53 mm, [45,48,50]) with a wide distribution throughout the eastern parts of southern Africa and along the south western coastal belts (Fig 2) [48], extending up through Africa to the Mediterranean [51]. There is geographical variation in echolocation frequencies across its range. In southern Africa, *R*. *clivosus* emits FM-CF-FM echolocation pulses at a RF of 90 to 92 kHz [45] (“Rcl_92_”), whereas towards the northern extent of its range, *R*. *clivosus* echolocates at lower frequencies (84–87 kHz) [51,52].

*Rhinolophus blasii* is a smaller rhinolophid (FA: 46 mm, [48]) and is distributed widely but sparsely throughout the eastern parts of southern Africa and Europe, partly overlapping with the distribution of *R*. *clivosus* (Fig 2). Similar to *R*. *clivosus*, *R*. *blasii* emits FM-CF-FM echolocation pulses, the RF of which also varies across its range. In areas where *R*. *blasii* occurs without *R*. *clivosus*, *R*. *blasii* uses a RF which overlaps with that of *R*. *clivosus* (RF: 90–92 kHz “Rbl_92_”; [this study]). However, in areas where the two species co-occur, *R*. *blasii* uses lower frequencies of 85–87 kHz (“Rbl_87_” [53,54]).

*Rhinolophus capensis* (FA: 46–51.8 mm, [45]) is endemic to the Cape Floristic Region in the south Western Cape of South Africa, extending as far north as the border between South Africa and Namibia. Over most of its range, *R*. *capensis*, often occurs in sympatry with *R*. *clivosus* but does not co-occur with *R*. *blasii* (Fig 2). Similar to both *R*. *clivosus* and *R*. *blasii* but to a much greater extent, the RF used by *R*. *capensis* varies across its distributional range (75–87 kHz, “Rca_87_” [34,49]) thus the RF of some *R*. *capensis* populations overlap with that of Rbl_87_, but does not overlap with that of Rcl_92_.

The pattern of RFs across these three species allowed us to test whether *R*. *clivosus* can discriminate between its own pulses and those of congenerics with RFs which do not overlap with its own (Rcl_92_ versus Rca_87_ and Rbl_87_) and those of a congeneric (*R*. *blasii*) with overlapping (Rcl_92_ versus Rbl_92_) and discrete (Rcl_92_ versus Rbl_87_) RFs. It also allowed us to test the ability of *R*. *clivosus* to discriminate between the pulses of two congenerics with the same RFs (Rca_87_ versus Rbl_87_).

### Study sites

The study was conducted in two parts at different localities. The first part was conducted at an animal housing facility in the Department of Biological Sciences of the University of Cape Town (UCT) (January—February 2014). The second part was conducted in the field at a research station situated within De Hoop Nature Reserve in the south-Western Cape of South Africa (34^0^26’S, 20^0^25’E, Fig 2). The research station was situated within 10 km of De Hoop Guano Cave from which *R*. *clivosus* test subjects used in the playback experiments were captured (June 2014).

### Animal capture and husbandry

Bats were captured using hand nets inside De Hoop Guano Cave. The capture and handling complied with the guidelines recommended by the American Society of Mammologists [55] and Kunz and Parsons [56]. This research was conducted under a Cape Nature permit (AAA007-00113-0056) and Animal ethics clearance (2013/2012/V33/DJ) from the Animal Ethics Committee of UCT.

All captured bats were hand-fed daily with fortified mealworms to ensure high nutritional levels of the novel food source. The health and weight of each bat was assessed daily to ensure that only healthy bats that had stabilized in captivity would be used in the experiments.

At UCT, bats were housed in a large indoor enclosure (3.3 m x 3 m x 2 m), lined with 5 mm mesh polyethylene netting. Temperature within the room was controlled at 24°C ± 2°C. Humidity was maintained between 50% and 80%. Day-night cycles were controlled artificially using red lights connected to an automated timer switch. Cycles were shifted by an hour a day until night day cycles were inverted. Bats had free access to water and were fed daily. After the period of captivity, bats were inspected by a registered veterinarian before being released at the site of capture.

### Experimental setup and procedure

Habituation-dishabituation experiments [57] were conducted in a sound attenuating box. The setup was the same as in Bastian & Jacobs [34] which is based on that in Schuchmann & Siemers [2]. Playbacks were produced using an ultrasound speaker (Avisoft UltraSoundGate Player BL Light, Avisoft Bioacoustics, Glienicke, Germany). Behavioural responses were recorded using an infrared video camera (Bell + Howell DNV16HDZ, New York, USA), while acoustic responses were recorded using an ultrasound microphone (Avisoft UltraSoundGate 416, Avisoft Bioacoustics, Glienicke, Germany) and Avisoft-Recorder software (Version 4.2, Avisoft Bioacoustics, Glienicke, Germany) on a Lenovo Ideapad laptop (Model Y580). An external monitor was connected to the video camera to allow real-time observations of the experiments during the trials. Video and audio recordings were synchronised using the terminal white noise control, which was visible on the audio line of both the video and audio software. For this, the recordings were aligned at the point of the white noise and the timing of the onset of the test signal was measured backwards from there.

Bats were placed on the perch inside the box and allowed time to habituate to the sound-attenuating box in silence. Habituation was defined as a point where a bat is silent and immobile at the perch for at least 20 s [34]. Immobility included no crawling activity, no ear movements, no head movements and no stretching of wings or legs. Once habituated to the box, the habituation playback was presented to the bat continuously, and the bat was allowed to habituate to this playback. When the bat showed no activity for 20 s, i.e. was habituated, the playback was changed to the test playback. There was a pause of approximately 150 ms as a result of the changeover from habituation to test playbacks. This delay was due to computer processing of the playback and unavoidable. The habituation file was placed on a loop in the event that the bat took longer than 300 s to habituate (which was the duration of an individual playback file). Trials were stopped if the bat was not habituated after 60 min. Such trials were excluded from the analysis and the trial was repeated on another night.

Experiments were divided into two batches, described henceforth as Experiment One and Experiment Two. Experiment One refers to those conducted at the UCT animal housing facility that tested the ability of bats to discriminate between species. Experiment Two refers to those conducted at the field research station that tested the ability of bats to discriminate between individual pulse components. Each experimental trial contained a single playback combination, consisting of one habitation playback and one test playback. For both experiments, each test individual was presented with all playback combinations associated with the respective experiment. For Experiment One, each bat was presented with a single trial per day over five consecutive days. For Experiment Two, each bat was presented with either two or three trials per day, with all trials spread across two days. The order in which bats were tested was randomised daily resulting in each bat being tested at a different time each night. The order of presentation of playback classes was also randomised (resulting in each playback combination being presented at a different time slot each day) to avoid any effect of trial order on behavioural responses to any particular individual or playback category.

#### Preparation of playback files

Playback stimuli were prepared using pulses from an existing database (compiled by DSJ) of echolocation sequences which were recorded from captured hand-held bats using the Avisoft UltraSoundGate 416 (Avisoft Bioacoustics, Glienicke, Germany), with a sampling rate of 500 kHz or the Pettersson D1000X (Pettersson Elektronik, Uppsala, Sweden) with a sampling rate of 340 kHz. We selected only pulses with similarly good quality (i.e. high signal-to-noise ratio) and avoided the first five pulses in a sequence because horseshoe bats are known to tune into their frequency after a period of silence [58,59]. To reduce unwanted noise or recording artefacts influencing the bat’s response to the playbacks, semi-synthetic duplicates of all selected pulses were produced in Avisoft-SASLab Pro (v.5.2.07, Avisoft Bioacoustics, Glienicke, Germany) using the variables of the dominant second harmonic of the original pulse. We created playbacks from echolocation pulses of the different populations/species which then represented the different stimuli classes in Experiment One and created playback stimuli classes based on each of the systematic manipulations on the average pulse template for Experiment Two. Playbacks were categorised into one or both of two configurations. The first was a habituation playback (duration = 300 s) used to habituate the test subject to that specific class of pulse. The second was a test playback (duration = 20 s), used to dishabituate the bats from the initial habituation pulse to test their ability to discriminate between classes. We applied an automated normalisation function using the “Normalize” option in the dialog box “Edit > Change volume” (Avisoft-SASLab Pro, v.5.2.07) which adjusts the intensity of all pulses to the same maximum i.e. to the same proportion of the recorded voltage. This results in pulses of a constant intensity within and across the playbacks and allowed us to control for variability in pulse intensity. Pulse intensities were normalised to a constant intensity using an automated normalisation function (Avisoft-SASLab Pro, v.5.2.07) within and across all playbacks. Output intensities were controlled using Avisoft Recorder (Avisoft Bioacoustics, Glienicke, Germany) and amplifier volume and gain were kept constant for all trials. Intensity was set according to recordings of live bats made inside the experimental box. Test playbacks were terminated with a 0.02 s broadband white noise as a control for false negative responses (i.e. bats having fallen asleep or experimental fatigue) [60]. A control trial in which both the habituation and test signals were the same class was used to control for false positives. A fade-in from 0 dB to maximum intensity was used across the duration of the first pulse in each playback file, to avoid crackling when the speaker broadcasts at a sudden high intensity.

### Experiment one–Species discrimination

To test the ability of bats to discriminate between different species at discrete and at overlapping frequencies of echolocation pulses, we used a playback experiment incorporating four playback stimuli classes: Rcl_92_, Rbl_92_, Rbl_87_, and Rca_87_. Recordings from *R*. *blasii* were split into two classes, based on different populations using discrete ranges of resting frequencies (Fig 2)–one population of *R*. *blasii* echolocating from 86–88 kHz (Rbl_87_ –Gatkop Cave, South Africa) and another from 91–93 kHz (Rbl_92_ –Kalenda, Zambia). A single class from each of the other two species, *R*. *clivosus* (Rcl_92_ –De Hoop Nature Reserve, South Africa) and *R*. *capensis* (Rca_87_ –Table Farm, South Africa) was created. As the test subjects were Rcl_92_ from De Hoop, the habituation file consisted of pulses from Rcl_92_ individuals which were different pulses than the ones used in the control Rcl_92_ test playback file. For the “foreign-foreign species, overlapping frequencies” playback combination (Table 1) the habituation class consisted of pulses from Rca_87_.

**Table 1:** Playback combinations for Experiment one.

Playback Combination	Habituation class	Test class
Control combination; own-own species, same frequencies Sympatric	Rcl_92_	Rcl_92_
Own-foreign species, overlapping frequencies Allopatric	Rcl_92_	Rbl_92_
Own-foreign species, discrete frequencies Sympatric	Rcl_92_	Rbl_87_
Own-foreign species, discrete frequencies Sympatric	Rcl_92_	Rca_87_
Foreign-foreign species, overlapping frequencies Allopatric	Rca_87_	Rbl_87_

For each of the four test playback stimuli classes we selected a set of 200 echolocation pulses (n) from different individuals (N) (Rcl_92_, N = 20, n = 10; Rbl_92_, N = 6, n = 34; Rbl_87_, N = 10, n = 20, Rca_87_, N = 20, n = 10). To avoid pseudo-replication, equal numbers of pulses from both male and female individuals were used in the playback recordings to preclude test subjects discriminating on the basis of gender instead of species [61]. Similarly, the use of pulses from multiple individuals precluded individual-based discrimination. Finally, we used multiple pulses per individual to ensure a representative sample of each individual’s pulses. To further minimise the effect of individual recognition rather than species discrimination, the semi-synthetic pulses were compiled into playback files in random order. Inter-pulse intervals (IPI) separating the pulses were based on the IPI preceding the pulse in the original recorded sequence.

The four different playback classes were grouped into five playback combinations (Table 1) in which the first class represented the habituation class and the second class represented the test. Playback combination included one control and four test combinations as follows: i) Rcl_92_-Rcl_92_ (control combination; own-own species, same frequencies); ii) Rcl_92_-Rbl_92_ (own-foreign species, overlapping frequencies); iii) Rcl_92_-Rbl_87_ (own-foreign species, discrete frequencies); iv) Rcl_92_-Rca_87_ (own-foreign species, discrete frequencies); and v) Rca_87_-Rbl_87_ (foreign-foreign species, overlapping frequencies). Bats were habituated with the first playback class and tested with the second.

The multiple pulse variables (S1 Table) of the playback files (Rcl_92_, Rbl_92_, Rbl_87_, Rca_87_) were measured and analysed (using the automatic parameter measurement function in Avisoft-SASLab Pro v.5.2.07, Avisoft Bioacoustics, Glienicke, Germany) to determine the acoustic variation between playback classes. To verify the correct measurement of frequency and duration variables we randomly measured 50 pulses manually from the power spectrum or the oscillogram respectively.

### Experiment two–Acoustic discrimination cues

To explore which components of echolocation pulses have the potential to encode discrimination cues, we used playback sequences compiled from fully synthetic pulses, in which we varied one acoustic component at a time while keeping all others constant. We used mean values measured in natural *R*. *clivosus* pulses (N = 20 (10 males; 10 females); n = 10 per individual; location: De Hoop Nature Reserve) for the variables resting frequency, bandwidth, sweep rate of FM components, duration of CF component, and IPI, to produce a synthetic average call (designated “Control call”). Based on this average call template we created six pulse variants each differing in only one specific pulse parameter. Three variables of echolocation pulses were altered, namely: i) frequency of the CF component; ii) duty cycle–the ratio between the duration and IPI of a string of pulses; and iii) sweep rate of the FM component. The pulse variant in which the frequency of the CF component was altered (designated “Frequency”) was created by decreasing the frequencies of the CF component and the corresponding FM components of the Control pulse by 5 kHz, maintaining the sweep rate and duty cycle of the control pulses. Pulse variants designated “FM1” and “FM2” were compiled by changing the sweep rate of the flanking FM components of the Control pulse, maintaining values within the natural range of observed pulses (BW_i_: 5–15 kHz; BW_t_: 5–28; D_i_: 1–4.4 ms; D_t_: 1.8–4.8 ms). FM1 had a lower sweep rate and FM2 a higher sweep rate. Both the initial and terminal FM components were altered in the same way for each of FM1 and FM2. The pulse variants “Duty Cycle1” and “Duty Cycle2” were compiled by altering the duration of the CF component of the pulses. In Duty Cycle1 the duration of the CF component was set to the maximum naturally observed duration, decreasing the period and increasing the duty cycle in sequences of these pulses. Accordingly, in Duty Cycle2, the durations of the CF components were set to the minimum naturally observed duration increasing the period of the pulses and lowering the duty cycle in sequences of these pulses.

Playback files were compiled by repeating a single pulse variant (Control, Frequency, FM1, FM2, Duty Cycle1, Duty Cycle2), maintaining an IPI equal to the average of naturally recorded intervals, until the required duration of the playback file was reached. The playback files were assigned to four playback combinations each containing a habituation playback of 300 s and a test playback of 20 s: 1: Control–Control, 2. Control–Frequency, 3. FM1 –FM2, 4. Duty Cycle1 –Duty Cycle2.

### Behavioural responses–Video analysis

The behavioural response of the bats listening to the playbacks was measured from video recordings for 20 s at three timeframes across each trial: onset of habituation (HabSTART), final period of habituation before playback was changed (HabEND) and during the test playback period (TEST). The observers rating the behavioural responses of the bats from the videos did so without any information regarding the trials (i.e. which test playback was used) and their ratings were not therefore influenced by prior knowledge of the trials and their expected outcomes. To control for false negative discrimination responses (e.g. experimental fatigue), only trials in which the bat reacted to both the onset of habituation and the terminal white noise were considered successful and therefore included in the analyses.

The Solomon Coder (v.14.05.18, András Péter, Hungary, http://www.solomoncoder.com) was used to record the duration and frequency of occurrence of 17 behaviours (S2 Table) that were observed during video analysis of captive *R*. *capensis* held at the animal housing facility at UCT [20]. Individual behaviours were categorised as either attentive or inattentive. Attentive behaviours were defined as behaviours associated with listening, scanning the environment or echolocating as well as behaviours observed in direct response to external stimuli (e.g. sudden loud noise or experimental stimuli). Inattentive behaviours, on the other hand, were defined as behaviours that were not performed in response to an external or experimental stimulus, e.g. stretching, grooming, etc. Statistical analysis focused on the eight attentive behaviours: Rapid Ear Twitching (RET), Slow Ear Twitching (SET), Full Head Lift (FHL), Partial Head Lift (PHL), Partial Leg Contractions (PLC), Full Leg Contractions (FLC), Scan (SC) and Echolocation Pulses (EC). For the statistical analyses the durations of all above listed attentive behaviours were combined (referred to as “Attentive”). As single behaviours could overlap in time, we avoided simply adding the durations, preferring instead to discard overlapping behaviours. The overall strength of a behavioural response was defined using duration of the combined attentive behaviours without overlap.

### Statistical analyses

#### Experiments one & two

The same procedure of statistical analysis was followed for both experiments. All statistical testing was conducted using Statistica 64 (Version 11, StatSoft Inc., Tulsa, USA) with a significance level of α = 0.05.

Shapiro-Wilks tests, which are efficient with smaller sample sizes, were used to test for normality of the data to determine if non-parametric tests should be used. A Friedman ANOVA was then used to analyse the potential long-term effects of the experimental repeats over multiple nights. For these tests, the durations of attentive behaviours during the HabSTART phase were used across all trials. To verify that bats were indeed fully habituated to the habituation stimulus before playing back the TEST stimulus, the duration of attentive behaviours during HabSTART and HabEND were compared for each playback combination using Wilcoxon Pairs Tests. To test if bats discriminated between the habituation playback and the test playback, the duration of attentive behaviours during HabEND and TEST were compared for each playback combination using Wilcoxon Pairs Tests.

To test if the ability of bats to discriminate between stimuli differed between playback combinations, the number of trials in which the bats discriminated the test playback from the habituation playback was compared between all playback combinations using a Pearson Chi Squared Test. Post hoc pairwise comparisons were conducted using Fisher Exact test to examine the number of successful discriminations between all permutations of the five playback combinations. Corrections for multiple comparisons were not applied during post hoc tests due to the impact of such manipulations on statistical power, particularly of analyses involving small sample sizes [62].

To test if there was a difference in the strength of response between playback combinations, the duration of attentive behaviours in response to the test playback was compared using a Kruskal-Wallis ANOVA, with all post hoc multiple pairwise comparisons.

Inter- and intra-observer reliability tests were used to test for potential observer bias in video analysis. Ten videos were selected at random and re-coded by an external observer or for the intra-observer reliability test by the same observer, and the results were compared with those of the original coding using Kappa Coefficients for frequency data and Wilcoxon Pairs Test for duration.

#### Acoustic characterization of playback classes

Pulse variables of the playback files (S1 Table) were analysed using Discriminant Function Analysis (DFA), followed by Canonical Correlation Analysis to characterise the acoustic variation between playback classes (Experiment One: Rcl92, Rbl92, Rbl87, Rca87). A correlation matrix–using a Pearson product-moment correlation coefficient–used to examine the correlation between call variables. This was followed by a principle component analysis (PCA) which was used to extract a set of non-correlated variables for the DFA from the set of highly correlated echolocation variables (defined by significant Pearson product-moment correlation). The Kaiser criterion of eigenvalues over 1 was used to select principle components for use in the DFA.

## Results

### Can bats discriminate between species?

Twelve male *R*. *clivosus* individuals were tested, from which we obtained a total of 49 successful trials (i.e. bats responded to both the onset of habituation and the terminal motivation control). A total of 11 trials were unsuccessful (bats did not respond to either the onset of habituation or the terminal control) and were excluded from the analysis. Both inter- and intra-observer biases were discounted and a high agreement between observers was confirmed (Kappa Coefficient: Inter: K = 0.87, N = 10, p > 0.05; Intra: K = 0.85, p > 0.05. Wilcoxon Pairs Test: Inter: Z = 0.62, p > 0.05; Intra: Z = 0.64, p > 0.05). The durations of attentive behaviours were non-normally distributed for all three phases within experimental trials (Shapiro-Wilk: HabSTART W = 0.84, p < 0.001; HabEND W = 0.29, p < 0.001; TEST W = 0.92, p < 0.005), therefore non-parametric tests were used for subsequent analysis.

The duration of attentive behavioural responses did not differ significantly between sequential trials for any of the three experimental phases (HabSTART, HabEND, TEST), suggesting that the order in which playback combinations were presented across different nights did not influence the bats ability to discriminate between playbacks (Friedman Test: HabSTART χ2 = 3.86, *df* = 4, p > 0.05; HabEND χ2 = 3.5, *df* = 4, p > 0.05; TEST χ2 = 3.69, *df* = 4, p > 0.05).

**Fig 3:**
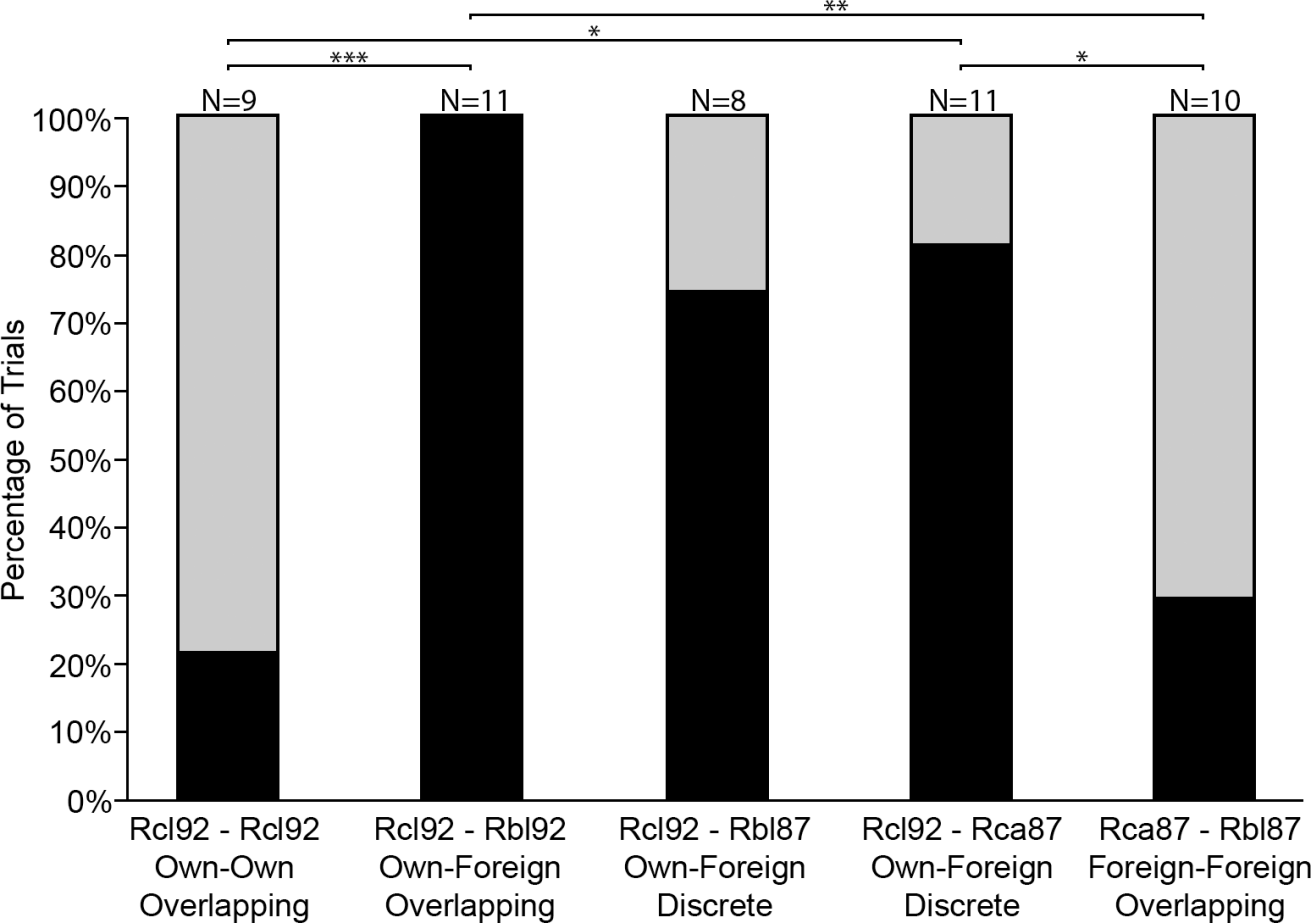
Discrimination of echolocation pulses of different *Rhinolophus* species by *Rhinolophus clivosus*. Black indicates the percentage of trials in which the test subjects discriminated between habituation and test playbacks. Grey indicates trials in which test subjects generalised habituation and test playbacks as belonging to the same class of stimuli. Lines with asterisks indicate significant outcomes from post hoc comparisons (Fisher exact test, *: p < 0.05, **: p < 0.005, ***: p < 0.0005); N is the number of trials.

The number of trials in which bats were able to discriminate between the habituation and test playback differed significantly between the five playback combinations (χ^2^ = 10.91, *df* = 4, p < 0.05; Fig 3). Post hoc comparisons revealed that in two of the four playback combinations involving foreign species, there were significantly more trials in which individuals responded to the test playback, i.e. discriminated own from foreign pulses, than to the control Rcl_92_-Rcl_92_ (Rcl_92_-Rbl_92_, Fisher Exact, p < 0.0005; Rcl_92_-Rca_87_, Fisher Exact, p < 0.05) (Fig 3). The number of trials in which individuals that discriminated between own-foreign, and discrete-sympatric (the Rcl_92_-Rbl_87_ combination) was not significantly different from that in the control at the 95% confidence level but was significant at the 94% confidence level (Fisher Exact, p = 0.056), with bats discriminating in 6 out of 8 trials between Rcl_92_ and Rbl_87_. When presented with playbacks from two foreign species, allopatric with respect to each other, with overlapping frequencies (Rca_87_-Rbl_87_), in only three out of ten trials did individuals discriminate, which did not differ significantly from the control (Fisher Exact, p > 0.05; Fig 3). Thus *R*. *clivosus* was able to discriminate between con- and heterospecifics, with both overlapping and discrete frequencies, but the ability to discriminate is greatly reduced when presented with similar pulses from two foreign species.

**Fig 4:**
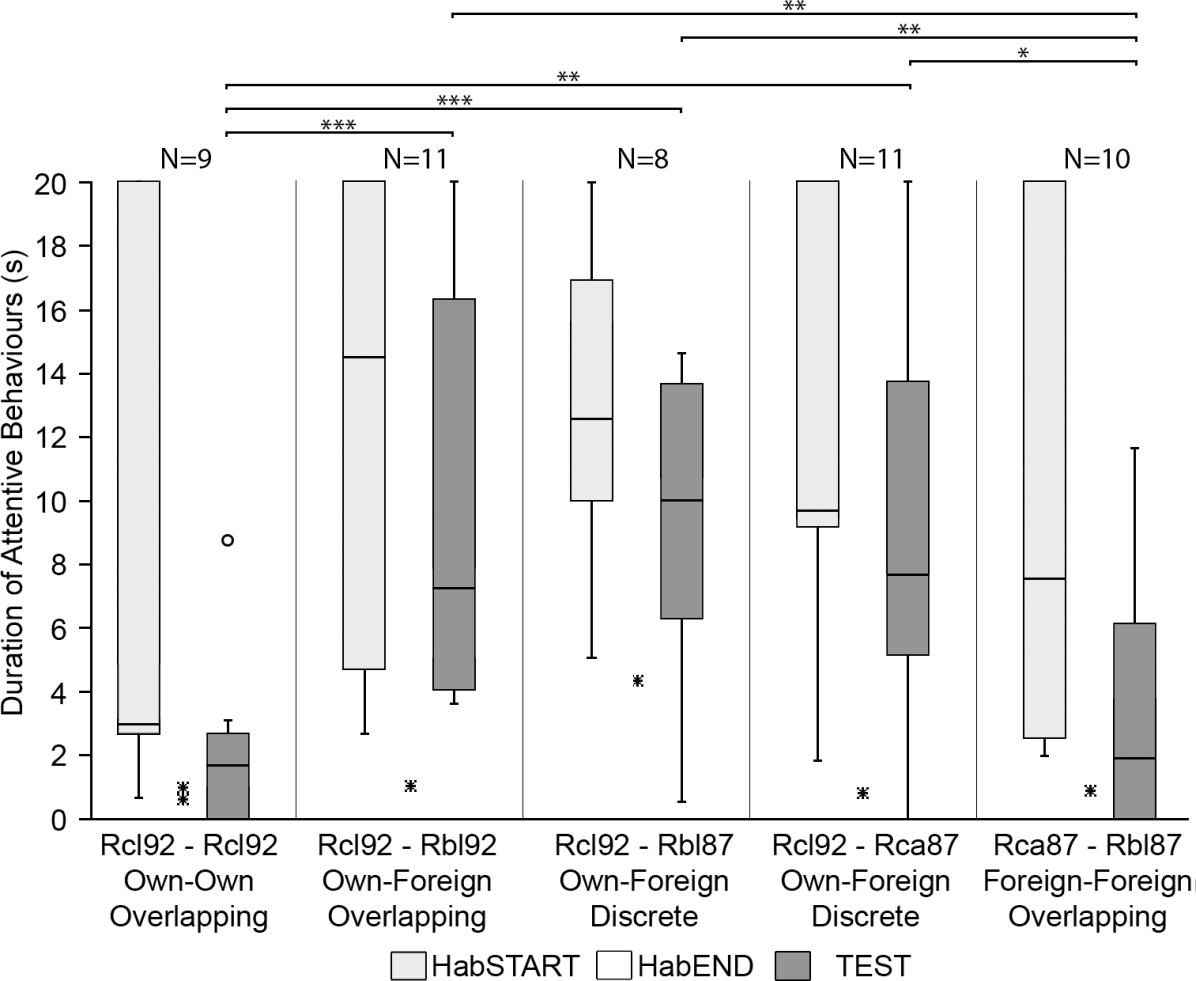
Durations of attentive behaviours during the three analysed experimental phases (HabSTART = light grey, HabEND = white and TEST = dark grey) for each of the four species discrimination trials. Boxes represent the interquartile range, horizontal lines represent the median, whiskers show non-outlier range within 1.5 times of the interquartile range, outliers outside 1.5 times the interquartile range are presented by circles and extremes (over twice the interquartile range) represented by asterisks. The durations of attentive behaviours for the HabEND phase were close to zero and is just visible on this graph. Lines and asterisks indicate outcomes from all possible post hoc comparisons between the TEST phases (*: p < 0.05, **: p < 0.01, ***: p < 0.001); N is the number of trials.

The duration of attentive behaviours was significantly shorter during the HabEND phase (Fig 4) than during the initial onset of HabSTART (Fig 4) for all playback combinations (Wilcoxon Pairs Test: Control Rcl_92_-Rcl_92_, *Z* = 2.52, p < 0.05; Rcl_92_-Rbl_92_, *Z* = 2.93, p < 0.005; Rcl_92_-Rbl_87_, *Z* = 2.52, p < 0.05; Rcl_92_-Rca_87_, *Z* = 2.93, p < 0.005; Rca_87_-Rbl_87_, *Z* = 2.80, p < 0.01), suggesting that habituation was successful.

The duration of attentive behaviours observed during the TEST phase (Fig 4) was significantly longer than that observed in the HabEND phase for all trials involving discrimination of heterospecifics from their own echolocation pulses (own-foreign), regardless of the frequency of the playback (Wilcoxon Pairs Test: Rcl_92_-Rbl_92_, *Z* = 2.93, p < 0.005; Rcl_92_-Rbl_87_, *Z* = 2.52, p < 0.05; Rcl_92_-Rca_87_; Fig 4) and for the foreign-foreign combination (*Z* = 2.66, p < 0.01; Rca_87_-Rbl_87_, *Z* = 2.02, p < 0.05; Fig 4). In contrast, the control playback combination (Rcl_92_-Rcl_92_) showed no significant difference between the HabEND and TEST phases (Wilcoxon Pairs Test, Control, *Z* = 1.78, p > 0.05), indicating that the effect of false positive responses by the bats are negligible and that observed behavioural responses are indicative of discrimination between habituation and test playbacks.

Furthermore, the duration of attentive behaviours during the TEST playback differed significantly between playback classes (Kruskal-Wallis, *H (4)* = 18.42, p < 0.01). Post-hoc multiple pairwise comparisons between playback combinations showed the duration of attentive behaviours in the TEST for all own-foreign combinations (Rcl_92_-Rbl_92_, Rcl_92_-Rbl_87_ and Rcl_92_-Rca_87_) were significantly higher than for the control, whereas the duration of attentive behaviours was not significantly higher for the foreign-foreign (Rca_87_-Rbl_87_) combination (Fig 4).

The longest attentive behavioural responses occurred in trials in which bats were presented with pulses from their own versus a foreign species (Fig 4). The test of Rcl_92_-Rbl_87_ combination yielded a response duration of 10.4 s (median), followed closely by that of the Rcl_92_-Rbl_92_ combination with 7.3 s and that of the Rcl_92_-Rca_87_ combination with 7.8 s, although there was no significant difference in response duration between the three (Post Hoc comparisons: p > 0.05, Fig 4). The control trial yielded the lowest duration of response with a duration of 1.8 s. The duration of response for the Rca_87_-Rbl_87_ combination was only slightly stronger at 1.9 s, with no significant difference between the two (Post Hoc comparisons: p > 0.05, Fig 4).

#### Acoustic characterization of playback classes

Examination of a correlation matrix revealed significant correlations between all but three acoustic call parameter pairings (Pearson product-moment correlation: N = 932, p < 0.05). A principal component analyses (PCA) was used to extract a set of ten linearly uncorrelated variables from variables measured for all 926 pulses across the four playback test classes. The first four principal components (PC 1–4) had Eigenvalues ≥ 1 [63] and accounted for 79.23% of the variation and were therefore used for further analysis (S3 Table). The bandwidths of the initial and terminal FM components (BW_i_ and BW_t_), as well as the sweep rate in these two components (SR_i_ and SR_t_) loaded highest on PC1. Pulse duration (D), inter-pulse interval (IPI) and the duration of the initial FM component (D_i_) loaded highest on PC2. Peak and maximum frequencies (RF and F_max_) as well as the bandwidth of the terminal FM component (BW_t_) loaded highest on PC3. The duration of the terminal FM component (D_t_) loaded highest on PC4 (S3 Table).

**Fig 5:**
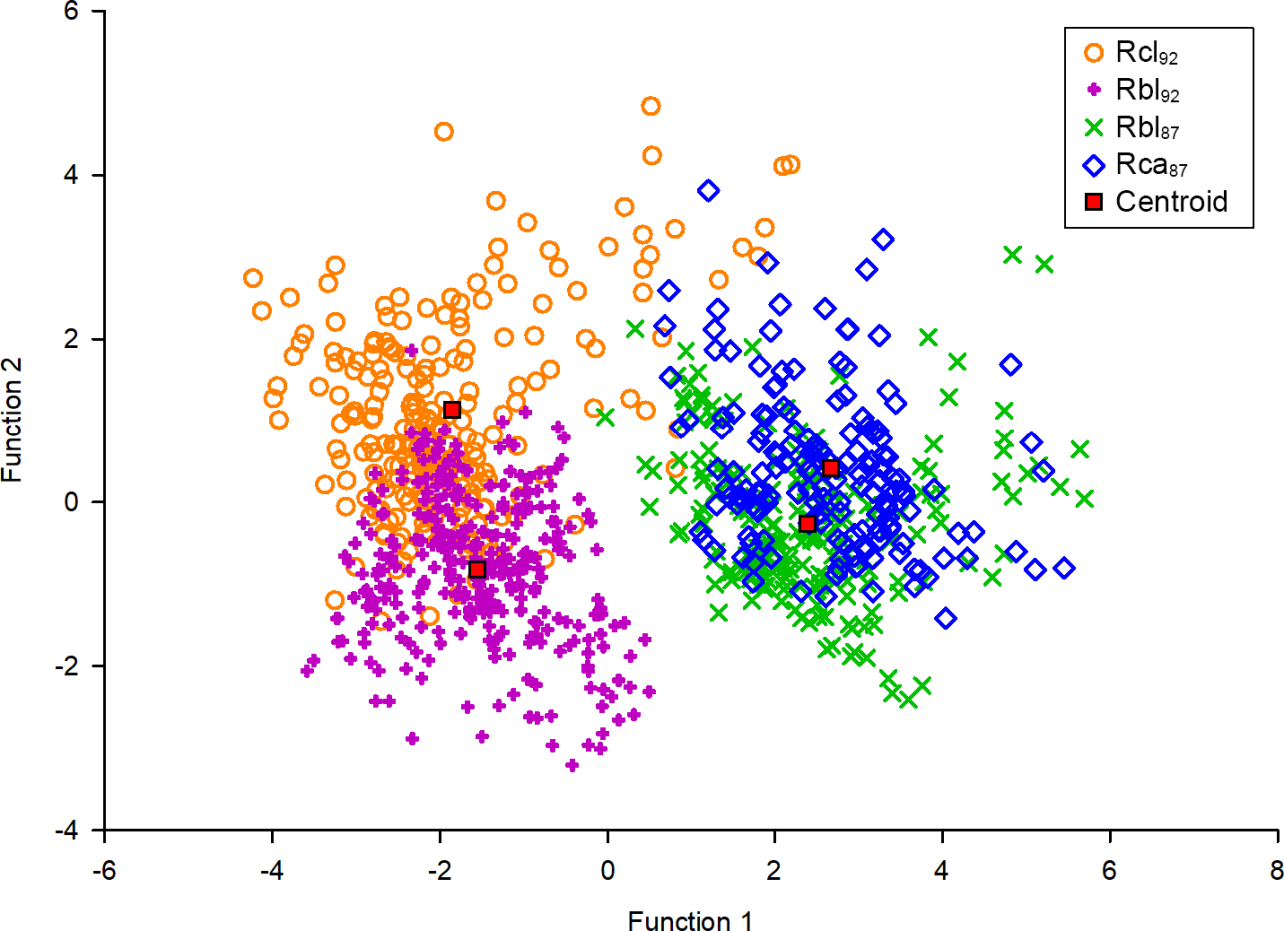
Plot of the first two functions of a DFA on ten acoustic variables of the four playback stimuli. Each pulse of each playback class is plotted (n = 926) and each playback class is colour coded. Function 1 represents mainly spectral variables and Function 2 represents mainly temporal variables.

The four PCs were used as variables in a discriminant function analysis (DFA). The DFA showed significant separation between the four playback classes based on their acoustic characteristics (Wilks *λ* = 0.102, F_12,2437_ = 277.37, p < 0.001) and all four playback combinations differed significantly from one another (S4 Table) despite some overlap in acoustic space (Fig 5). The first discriminant function (Function 1) explained 85.1% of the variance and Function 2 explained a further 10.8% (Fig 5). Function 1 contained high loadings of PC1 and PC3 and its high discriminatory power is therefore mainly characterised by spectral variables of both the CF and the FM components (RF, F_max_, BW_i_ and BW_t,_) and sweep rate (SR_i_ and SR_t_). Function 2 which had much lower discriminatory power contained PC2 and PC4 and thus is characterised by temporal variables (D, D_i_, D_t_, and IPI).

The graphical plot of those two functions reveals the four playback classes formed two primary clusters separated along Function 1, (comprised of spectral variables; Fig 5). One primary cluster (in the right half of Fig 5) was comprised of pulses from the two 87-kHz classes (Rbl_87_, Rca_87_) and the other primary cluster was comprised of pulses from the two 92-kHz classes (Rcl_92_, Rbl_92_). All Mahalanobis distances between group centroids were >16 with the exception of those between the two 87-kHz classes (Rbl_87_ and Rca_87_) and the two 92-kHz classes (Rcl_92_ and Rbl_92_) which were less than 4 (S5 Table). However, pulses between the two 92-kHz classes were separated along Function 2 (temporal variables; S3 Table) and differed by Mahalanobis distance of 3.7, over 50% more than the distance between the two 87-kHz classes also separated along Function 2 (Fig 5). Function 2 was comprised of temporal variables (S3 Table).

Accordingly, pulses were correctly classified into their a priori assigned class (Rcl_92_, Rbl_92_, Rbl_87_ and Rca_87_) based on their acoustic variables with a success rate of > 80%. However, Rca_87_ was an exception with a lower classification success of 65% and most misclassifications in this class involved the pulses of the heterospecific Rbl_87_.

### Which cues, in addition to RF, are bats using to discriminate between conspecifics and heterospecifics?

Thirty-one *R*. *clivosus* individuals were tested (10 males, 21 females) with the four playback combinations (1: Control–Control, 2. Control–Frequency, 3. FM1 –FM2, 4. Duty Cycle1 –Duty Cycle2), from which we obtained 92 successful trials. A total of 36 trials were unsuccessful (the bats in these trials did not respond to either the onset of habituation or the terminal control signal) and were excluded from the analysis. The behavioural responses of males and females to the test playbacks did not differ significantly and results were thus pooled (χ^2^ = 0.0003, *df* = 1, p > 0.05). The durations of attentive behaviours were found to be non-normally distributed for all analysed phases (Shapiro-Wilk: HabSTART W = 0.88, p < 0.0001; HabEND W = 0.13, p < 0.0001; TEST W = 0.79, p < 0.0001). The strength of behavioural responses did not differ significantly between sequential trials for any of the three experimental phases, suggesting that the order in which playback combinations were presented to an individual did not influence the bats ability to discriminate between playbacks (Friedman Test: HabSTART χ^2^ = 1.27, *df* = 3, p > 0.05; HabEND χ^2^ = 3.44, *df* = 3, p > 0.05; TEST χ^2^ = 9.4, *df* = 3, p > 0.05). Successful habituation to the habituation stimulus was confirmed by significantly lower durations of attentive behaviours during the HabEND phase than during the HabSTART for all playback combinations (Wilcoxon Pairs Test: Control, *Z* = 4.01, p < 0.001;”Frequency”, *Z* = 4.29, p < 0.001; FM1, *Z* = 4.20, p < 0.001; duty cycle, *Z* = 4.20, p < 0.001).

**Fig 6:**
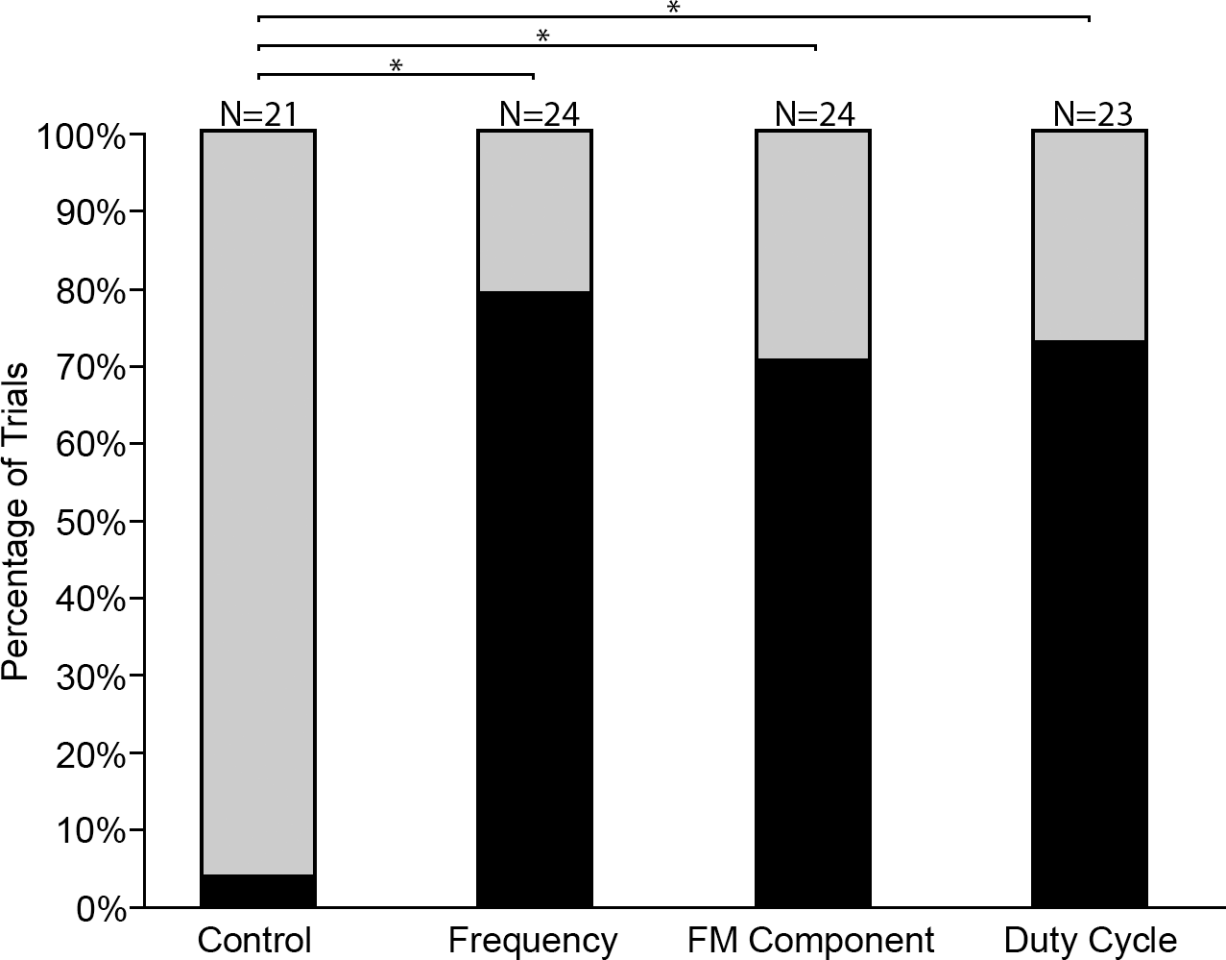
Discrimination of playback of individually manipulated echolocation pulse variables by *Rhinolophus clivosus*. Black indicates the percentage of trials in which the test subjects discriminated between habituation and test playbacks. Grey indicates trials in which test subjects generalised habituation and test playbacks as belonging to the same class of stimuli. Lines with asterisks indicate significant outcomes from post hoc comparisons (Fisher exact test, *: p < 0.0001); N is the number of trials. Key to the test combinations: Control = control-control; Frequency = control-frequency; FM component = FM2-FM2; Duty cycle = Duty cycle1-Duty cycle2.

The ability of bats to discriminate between the habituation and test playbacks differed significantly between the four playback combinations (χ^2^ = 42.46, *df* = 3, p < 0.001; Fig 6). Post hoc pairwise comparisons revealed that the number of trials in which individuals discriminated the test playback from the habituation playback for all three test playback combinations was significantly higher than that for the control (Fisher Exact: p < 0.0001 for all combinations, Fig 6). There were no significant differences in the number of trials in which bats discriminated between habituation and test signals among the test combinations (Control–Frequency, FM1 –FM2 and Duty Cycle1 –Duty Cycle2).

The control playback combination showed no significant difference in the duration of attentive behaviours between the HabEND and TEST phases (Wilcoxon Pairs Test, Control, *Z* = 0.54, p > 0.05), indicating that the observed behavioural responses are indicative of discrimination between habituation and test playbacks. All four test combinations yielded a significant increase in attentive behaviours in response to the test playback after habituation (Wilcoxon Pairs Test, Frequency: *Z* = 3.72, p < 0.001; FM: *Z* = 3.52, p < 0.005; Duty Cycle: *Z* = 3.12, p < 0.001).

**Fig 7:**
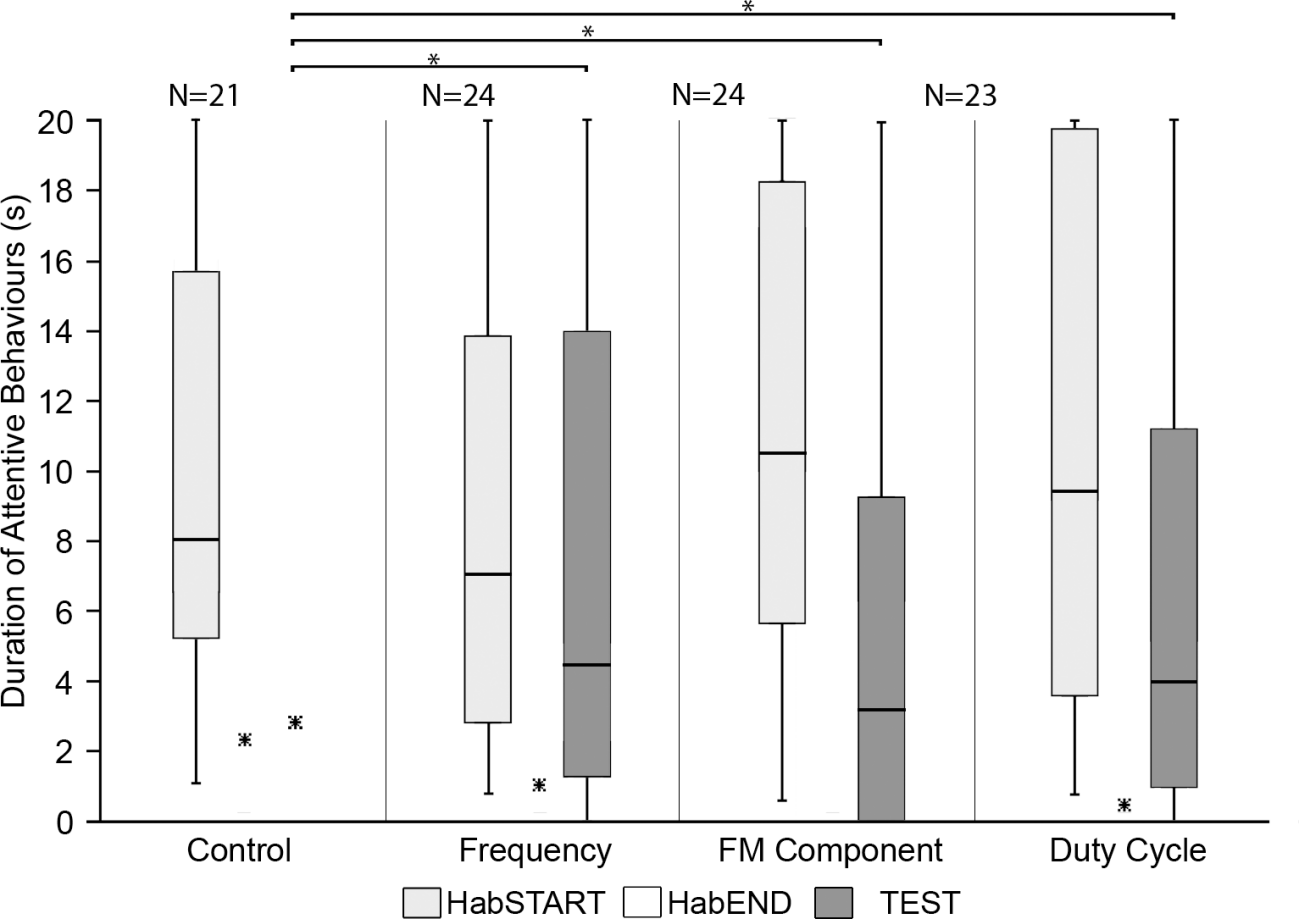
Durations of attentive behaviours during the three analysed experimental phases (HabSTART = light grey, HabEND = white and TEST = dark grey) for each of the four species discrimination trials. Boxes represent the interquartile range, horizontal lines represent the median, whiskers show non-outlier range within 1.5 times of the interquartile range, outliers outside 1.5 times the interquartile range are presented by circles and extremes (over twice the interquartile range) represented by asterisks. The durations of attentive behaviours for the HabEND phase were close to zero and is just visible on this graph. Lines and asterisks indicate outcomes from all possible post hoc comparisons between the TEST phases (*: p < 0.01); N is the number of trials.

The duration of attentive behaviours in response to the test playback differed significantly between playback combinations (Kruskal-Wallis, *H(3)* = 26.49, p < 0.001). Post-hoc multiple pairwise comparisons between individual playback combinations showed the duration of behavioural responses was significantly higher than the control for all three test combinations (Fig 7). There were however no significant differences among the three non-control test combinations (Fig 7).

The longest attentive behavioural responses were to changes in the frequency of the CF component (median = 4.3 s), followed by duty cycle (median = 3.9 s), and sweep rate of FM components (median = 3.2 s), although there was no significant difference in the durations of responses among the three tests. The control yielded no response (Fig 7).

## Discussion

As predicted by the ACH, bats were able to discriminate between its own pulses and that of the same congeneric whether the CF components had discrete or overlapping frequencies. When frequencies overlapped bats were nevertheless able to discriminate between classes by apparently using other variables of the echolocation pulses. Our study emphasises the importance of the multivariate acoustic space for the perception of vocal signatures in echolocation and identifies those acoustic components, in addition to resting frequency, that have the potential for use by bats to discriminate amongst congenerics.

### Can bats discriminate between species?

Horseshoe bats habituated with conspecific echolocation pulses and tested with echolocation pulses from a foreign species showed behavioural responses to the change in playback, demonstrating the ability to discriminate heterospecific pulses from those of conspecifics. This corroborates the findings of previous studies [2,31,34] that echolocating bats can discriminate between echolocation pulses of different species. Our study also shows that discrete frequency bands were not essential for discrimination of foreign species, as reported by some previous studies [2,34] and therefore supports the re-formulation of the ACH which emphasizes acoustic space rather than discrete frequency bands as a requisite for echolocation-based discrimination.

This is demonstrated here in the ability of bats to discriminate between playback classes that varied in their separation in acoustic space. For example, listening *R*. *clivosus* (Rcl_92_) could readily discriminate the echolocation pulses of Rbl_92_ from that of conspecifics, despite the overlap in frequencies. In this case, *R*. *clivosus* was able to discriminate probably based on differences in other pulse variables. In contrast, when two heterospecifics shared a similar acoustic space (Rca_87_ and Rbl_87;_ Fig 5), the ability of *R*. *clivosus* to discriminate between the echolocation pulses of these two species and those of conspecifics was greatly reduced, to the extent that the strength of response did not differ significantly from that of the control.

Unlike the echolocation pulses of Rca_87_ and Rbl_87_, Rbl_92_ and Rcl_92_ are separated to some degree by the temporal variables of pulse duration, duration of the initial and terminal FM components and the inter-pulse interval. The separation in temporal variables likely provides the variation in acoustic space required for effective species discrimination. However, there was nevertheless some overlap in these temporal variables and if this was the basis for successful discrimination, it suggests that bats may have considerable perceptual acuity. This supports the findings of a previous study on another rhinolophid, *R*. *capensis*, which reported differences in perceptual acuity among populations occupying assemblages that differed in acoustic complexity [34]. *R*. *capensis* populations in acoustically complex assemblages displayed greater discriminatory ability than *R*. *capensis* in acoustically less complex assemblages. The former was able to discriminate between different conspecific phonotypes and heterospecifics with very similar echolocation pulses to its own. In contrast, the latter, which was sympatric with only one other rhinolophid species (*R*. *damarensis*) which differed appreciably in pulse variables, was not able to discriminate between *R*. *damarensis* and conspecifics which had similar pulse variables [34]. Thus, the perceptual acuity displayed by bats may be adapted to the complexity of the acoustic environment in which they live. If so, it would explain why *R*. *clivosus* was unable to discriminate between Rca_87_ and Rbl_87_ but could readily do so between conspecifics (Rcl_92_) and Rbl_92_. Rca_87_ and Rbl_87_ are not sympatric and if this situation prevailed over the evolutionary history of *R*. *clivosus* it would not have been exposed to both Rca_87_ and Rbl_87_ in the same assemblage and therefore never evolved the perceptual acuity necessary to discriminate between these two phonotypes of *R*. *capensis* and *R*. *blasii*. Similarly, *R*. *capensis* and *R*. *blasii* are not sympatric anywhere in their ranges (Fig 2), thus there is no selective pressure for ‘private frequency bands’ [43] or discrete acoustic space between them to facilitate communication. In the absence of competition for acoustic space between *R*. *capensis* and *R*. *blasii*, similarities in their foraging ecology and habitat [48] likely result in the evolutionary convergence of both species on the same acoustic space in allopatry without compromising the communication potential in either species. The geographic distribution of a species in relation to similar congenerics (i.e. sympatric and allopatric) likely plays an important role in the evolution of both acoustic space and perceptual acuity.

The high perceptual acuity of *R*. *clivosus* allowing discrimination between Rcl_92_ and Rbl_92_ was a key difference between our study and that of Schuchmann and Siemers [2]. Testing a different community of horseshoe bats, Schuchmann and Siemers [2] found that the ability of *R*. *mehelyi* and *R*. *euryale* to discriminate heterospecific echolocation pulses was significantly lower when pulse frequencies overlapped than when they were discrete. However, in combination with the findings of Schuchmann and Siemers [2], the inability of *R*. *clivosus* to discriminate between two foreign heterospecifics suggests that selection pressure may be greater for discrimination between conspecifics and heterospecifics than for discrimination among different heterospecifics. This awaits confirmation through research on additional bat assemblages.

### Can bats perceive acoustic cues from multiple variables of echolocation?

*Rhinolophus clivosus* could successfully discriminate between synthesised echolocation pulses which differed in one of resting frequency, sweep rate of the FM components and the duty cycle of echolocation pulses. Much of the previous work on echolocation-based discrimination has focused on the frequency of the CF component (i.e. the resting frequency) in horseshoe bat echolocation pulses as a species-specific discrimination cue [2,31,34]. In discovering that bats could discriminate despite overlapping RFs, these studies revealed the potential for additional acoustic cues to influence discrimination when resting frequencies were similar [2,31,34]. Schuchmann and Siemers [2] speculated that the FM components of horseshoe bat pulses may encode such information. Bastian and Jacobs [34] later verified that a consideration of multiple variables resulted in bats occupying almost unique acoustic spaces. Our study provides experimental evidence that bats can perceive differences in acoustic variables separately from one another, supporting the notion of multidimensional acoustic space and its facility in species discrimination.

Of the three pulse components tested, changes in RF produced the highest proportion of trials in which bats successfully discriminated (Fig 6), suggesting that frequency provides a strong cue for species discrimination. However, both the FM components and duty cycle also yielded high rates of discrimination, demonstrating that these components likely offer important additional acoustic cues for species discrimination allowing bats to discriminate even when RFs overlap.

The different components of horseshoe bat echolocation pulses are complex in structure, with minor variations, for example, in the bandwidth, slope and sweep rate (see S2 and S5 Tables) of the FM components. Such variation provides potential, discrimination cues. Due to limitations in the number of trials with which each bat could be presented, only a single manipulation of the FM component could be used in our study. Nevertheless, bats could discriminate between pulses with different sweep rates–i.e. between pulses with a long, narrow bandwidth FM component and pulses with a short, broad bandwidth FM component. Even with just this change, bats were able to discriminate between playbacks, revealing sensitivity in the perception of this parameter. An aspect which deserves further attention is possible differences in function, in the context of species discrimination, between the initial and terminal FM components. Schuchmann and Siemers [2] as well as Bastian & Jacobs [34] placed special emphasis on the potential importance of the terminal FM component, which is often more prominent in the echolocation pulses of horseshoe bats. We manipulated both the initial and terminal FM components equally and simultaneously, although it is possible that a bat could vary these two components independently and that a listener could perceive these changes. Whether such manipulation by the sender, if it happens, facilitates or inhibits discrimination by the receiver remains to be discovered. The report of modified echolocation pulses in echolocation recordings of horseshoe bats [20], which enhances difference in acoustic space, provides a promising avenue for investigating the role of the FM component in discrimination.

The communicative potential of the FM component may also shed additional light on the overall function and evolution of the FM component in FM-CF-FM pulses. The FM component in horseshoe bat echolocation pulses functions in determining target distance [42,64,65]. The spectral and temporal features of the FM component have been shown to vary depending on a number of external factors, including the level of ambient noise [66] as well as the presence of other bats, both con- and heterospecifics [67]. The spectral features of the CF component are restricted by the acoustic fovea, limiting their ability to be adjusted to facilitate communication (but see [63,67,68]). In contrast, FM components may be altered in response to a number of external factors, and therefore have greater flexibility, as evidenced by modifications to it [20], to function in communication.

Changes in the duty cycle of playback sequences also elicited a strong behavioural response from the listening bats. The duty cycle of echolocation pulses has previously been shown to be of value to eavesdropping bats [69]. During foraging, the pulse rate and duration of echolocation pulses vary with each phase (search, approach and terminal) of an echolocation sequence [69], resulting in a ‘feeding buzz”. Eavesdropping bats have been shown to be attracted to pulse sequences which include feeding buzzes [69], demonstrating the potential value of changes in duty cycle to a listening bat. Our study, however, provides the first experimental evidence that a change in duty cycle alone, outside of the context of foraging, can elicit a behavioural response from a listening bat. This suggests a potential role in discrimination and possibly also in communication. The exact details of how the duty cycle of echolocation pulses influences discrimination remains unclear. Even from recordings of handheld bats, the duty cycle of echolocation pulses is observed to vary considerably, making it too variable to function as a reliable cue of species identify. Furthermore, the duty cycle playback consisted of a combination of two variables, inter-pulse interval and pulse duration and it is not clear if it is the pulse duration or the inter-pulse interval that drives this response, or if the two components necessarily need to work in conjunction with one another and with other components. Future studies should investigate the separate effects of these components to gain a better understanding of which components are important for discrimination. The effect on communication of changes in duty cycle during flight should also be considered.

## Conclusions

The ability of horseshoe bats to discriminate between species is not reliant on separation in frequency bands, but rather divergence in multidimensional acoustic space. Listening individuals can discriminate between the echolocation pulses of heterospecifics even when pulses share the same frequency band. This discriminatory ability is based on their ability to detect changes in multiple components of echolocation–including RF, FM components and duty cycle–which likely act in concert as acoustic cues for discrimination. Bats in the wild may be faced with constraints not present in the captive situation and whether bats use these echolocation components for species discrimination in the wild, remains to be determined.

## Supporting information

S1 Table List of pulse parameters measured for analysis of FM-CF-FM pulses. (DOCX)

S2 Table List of behaviours coded from video and/or audio records for during habituation-dishabituation experiments. (DOCX)

S3 Table Principle component loadings for the first four principle components extracted from the ten echolocation measurements. (DOCX)

S4 Table F-values obtained from Discriminant Function Analysis on echolocation call variables of four playback classes (*df* = 4; all p < 0.01). (DOCX)

S5 Table Squared Mahalanobis distances obtained from Discriminant Function Analysis on echolocation call variables of four playback classes. (DOCX)
