# Supplementary material for: It’s not all about the Soprano: Rhinolophid bats use multiple acoustic components in echolocation pulses to discriminate between conspecifics and heterospecifics
Source: PLoS One. 2018 Jul 18;13(7):e0199703. doi: 10.1371/journal.pone.0199703 (PMC6051568; doi:10.1371/journal.pone.0199703)
Supplement: S1 Table — (DOCX) [file pone.0199703.s001.docx]

**S1 Table:** List of pulse parameters measured for analysis of FM-CF-FM pulses.

| **Parameter** | **Abbreviation** | **Description** |
| --- | --- | --- |
| Maximum Frequency | F_max_ | Maximum frequency reached in the pulse. |
| Resting Frequency | RF | Frequency at the centre of the pulse. |
| Duration | D | Duration of entire pulse. |
| Inter-pulse interval | IPI | Duration of interval between the end of one pulse and the beginning of the following pulse. |
| Bandwidth of initial FM | BW_i_ | Range of frequencies represented in the FM component that precedes the CF component of a pulse. |
| Bandwidth of terminal FM | BW_t_ | Range of frequencies represented in the FM component that follows the CF component of a pulse. |
| Duration of initial FM | D_i_ | Duration of the initial FM component, measured from the start of the pulse to the point when frequency becomes constant. |
| Duration of terminal FM | D_t_ | Duration of the terminal FM component, measured from the end of the constant frequency component to the end of the pulse. |
| Sweep Rate of Initial FM | SR_i_ | Bandwidth divided by the duration of initial FM component. |
| Sweep Rate of Terminal FM | SR_t_ | Bandwidth divided by the duration of terminal FM component. |
