# Supplementary material for: It’s not all about the Soprano: Rhinolophid bats use multiple acoustic components in echolocation pulses to discriminate between conspecifics and heterospecifics
Source: PLoS One. 2018 Jul 18;13(7):e0199703. doi: 10.1371/journal.pone.0199703 (PMC6051568; doi:10.1371/journal.pone.0199703)
Supplement: S2 Table — (DOCX) [file pone.0199703.s002.docx]

**S2 Table**: List of behaviours coded from video and/or audio records for during habituation-dishabituation experiments.

| **Behaviour** | **Class** | **Description** | **Data Type** |
| --- | --- | --- | --- |
| Rapid Ear Twitching (RET) | Attentive | Continuous rapid movement of entire ear(s) towards sound source, single twitches cannot be counted in real time. | Duration |
| Slow Ear Twitching (SET) | Attentive | Continuous movement of entire ear(s) towards sound source, single twitches can be counted in real time. | Duration |
| Full Head Lift (FHL) | Attentive | Complete lifting of the head, to a point where the chin is visible, with no sideways movement or twitching of the ears. | Duration |
| Partial Head Lift (PHL) | Attentive | Partial raising of the head, to a point where the chin still cannot be seen. | Duration |
| Scan(SC) | Attentive | Continuous rapid movement of entire ear(s) towards the sound source and head fully lifted looking left and right. | Duration |
| Full Leg Contraction (FLC) | Attentive | Legs completely contracted by bending at the knee, often associated with hunching of body. | Duration |
| Partial Leg Contraction (PLC) | Attentive | Legs partially contracted by bending at the knee, often associated with hunching of body. | Duration |
| Echolocation Calls (EC) | Attentive | Count of echolocation pulses. | Count |
| Single Ear Twitch (SET) | Inattentive | Single movement of the ear(s). | Count |
| Tip of Ear Twitch | Inattentive | Single movement of only the tip of the ear. | Count |
| Head Swaying | Inattentive | Side to side movement of the head, without the head being lifted. | Duration |
| Body Shuffle | Inattentive | Side to side movement of the body without relocation of feet, usually ending in sleeping position. | Duration |
| Crawling | Inattentive | Repositioning along the perch, involving movement of feet and wings, settling in another position. | Duration |
| Vibrating Wings | Inattentive | Rapid vibrations of both wings, usually ending in a sleeping position. | Duration |
| Full Wing Stretch | Inattentive | Full length expansion of wing(s) to the side. | Count |
| Partial Wing Stretch | Inattentive | Partial expansion of wing(s) to the side. | Count |
| Grooming | Inattentive | Use of tongue of feet to groom fur and wings. | Duration |
