# Supplementary material for: It’s not all about the Soprano: Rhinolophid bats use multiple acoustic components in echolocation pulses to discriminate between conspecifics and heterospecifics
Source: PLoS One. 2018 Jul 18;13(7):e0199703. doi: 10.1371/journal.pone.0199703 (PMC6051568; doi:10.1371/journal.pone.0199703)
Supplement: S3 Table — (DOCX) [file pone.0199703.s003.docx]

**S3 Table:** Principle component loadings for the first four principle components extracted from the ten echolocation measurements.

| **Variable** | **PC 1** | **PC 2** | **PC 3** | **PC 4** |
| --- | --- | --- | --- | --- |
| Duration | -0.015 | **-0.762** | 0.342 | 0.073 |
| Inter-Pulse-Interval | 0.264 | **-0.624** | 0.346 | -0.182 |
| Resting Frequency | **-0.641** | 0.424 | 0.449 | 0.320 |
| Maximum Frequency | -0.436 | 0.530 | **0.633** | 0.185 |
| Bandwidth of initial FM | **-0.754** | -0.531 | 0.025 | -0.178 |
| Bandwidth of terminal FM | **-0.755** | -0.009 | -0.463 | 0.227 |
| Duration of initial FM | **-0.642** | -0.633 | -0.031 | -0.114 |
| Duration of terminal FM | -0.047 | -0.538 | -0.183 | **0.766** |
| Sweep Rate Initial FM | **-0.663** | -0.146 | 0.157 | -0.291 |
| Sweep Rate Terminal FM | **-0.677** | 0.413 | -0.328 | -0.210 |
