# Supplementary material for: It’s not all about the Soprano: Rhinolophid bats use multiple acoustic components in echolocation pulses to discriminate between conspecifics and heterospecifics
Source: PLoS One. 2018 Jul 18;13(7):e0199703. doi: 10.1371/journal.pone.0199703 (PMC6051568; doi:10.1371/journal.pone.0199703)
Supplement: S4 Table — (DOCX) [file pone.0199703.s004.docx]

**S4 Table**: F-values obtained from Discriminant Function Analysis on echolocation call parameters of four playback categories (df = 4; all p < 0.01).

| **Species/Class** | **Rcl92** | **Rbl92** | **Rbl87** | **Rca87** |
| --- | --- | --- | --- | --- |
| **Rcl92** |  | 123.4102 | 556.7822 | 513.3864 |
| **Rbl92** | 123.4102 |  | 527.8203 | 515.6454 |
| **Rbl87** | 556.7822 | 527.8203 |  | 53.3321 |
| **Rca87** | 513.3864 | 515.6454 | 53.3321 |  |
