# Supplementary material for: It’s not all about the Soprano: Rhinolophid bats use multiple acoustic components in echolocation pulses to discriminate between conspecifics and heterospecifics
Source: PLoS One. 2018 Jul 18;13(7):e0199703. doi: 10.1371/journal.pone.0199703 (PMC6051568; doi:10.1371/journal.pone.0199703)
Supplement: S5 Table — (DOCX) [file pone.0199703.s005.docx]

**S5 Table:** Squared Mahalanobis Distances obtained from Discriminant Function Analysis on echolocation call parameters of four playback categories.

| **Species/Class** | **Rcl92** | **Rbl92** | **Rbl87** | **Rca87** |
| --- | --- | --- | --- | --- |
| **Rcl92** |  | 3.70555 | 20.28414 | 21.79342 |
| **Rbl92** | 3.70555 |  | 16.41535 | 19.14054 |
| **Rbl87** | 20.28414 | 16.41535 |  | 2.32124 |
| **Rca87** | 21.79342 | 19.14054 | 2.32124 |  |
